# Social proximity in dairy calves is affected by differences in pessimism

**DOI:** 10.1371/journal.pone.0223746

**Published:** 2019-10-30

**Authors:** Benjamin Lecorps, Sarah Kappel, Daniel M. Weary, Marina A. G. von Keyserlingk

**Affiliations:** Animal Welfare Program, Faculty of Land and Food Systems, University of British Columbia, Vancouver, BC, Canada; University of Illinois, UNITED STATES

## Abstract

Negative social interactions have been extensively studied in dairy cattle, but little is known about the establishment of positive (preferential) relationships. Adult dairy cows are known to spend more time at close proximity to specific social partners, indicating that they establish stronger bonds with these animals, but few studies have explored what happens in socially housed calves. In this study, we explored whether calves that spent their entire life in the same social group established social preferences (i.e. pairs of individuals that interact more) that are stable over time (two 48-h periods, separated by three days), across two types of behavior (standing and lying) and across contexts (change in environment and housing design). When housed in an open pack, calves showed consistent proximity patterns when standing (but not when lying). These preferential relationships persisted even after calves were moved into a new pen fitted with free stalls. At the individual level, calves varied in how selective they were in their social relationships, with some calves spending much more time with specific partners than did others. This degree of selectivity was not associated to Sociability, marginally associated to Fearfulness, but was associated with Pessimism (more pessimistic calves were more selective in their social relationships). In conclusion, calves can form selective relationships that appeared to be consistent over time and across context, and the degree to which calves were selective varied in relation to individual differences in Pessimism.

## 1. Introduction

Social bonding has been found in many species [1] and enduring social connections between individuals, not directly related to mating, have important positive consequences including improved competitive ability, lowered stress and higher reproductive success. Bonding is usually found between close kin and has been traditionally observed in populations that contain different generations, such as wild or captive primates [2]. Little is known about social bonding in animals raised in artificial social groups such as dairy cattle.

There is increasing interest in understanding social dynamics in dairy herds [3–5]. Adult cows invest time and energy to secure social proximity to particular conspecifics, suggesting that they value these relationships [4]. However, little is known on younger animals. Dairy calves are typically separated from their mother hours after birth, and when socially housed they are often grouped with animals of similar age from birth to adulthood. Dairy calves are motivated to have full social contact [6] and develop social bonds with peers at an early age [7,8]. Social contact early in life has been associated with positive physiological, psychological and cognitive development in calves [9], but it remains unknown whether these animals develop preferential relationships.

Socially bonded individuals usually groom each other more and maintain close proximity [10]. Based on evidence that spatial proximity (measured as the number of times two animals were seen in close proximity) is associated with preferential relationships in adult cows [4,5,11,12], we used spatial proximity to assess social preference in dairy calves. We explored whether pairs of calves kept in social groups were consistent in their social preferences over time, across contexts (when moved to a new environment) and among behaviors (standing and lying). We predicted that pairs would vary in the degree that they interacted, and that these social preferences would be consistent across time, housing systems and behaviors.

Previous studies suggested that inter-individual differences in social relationships can be affected by personality traits. For instance, sociable animals are thought to “*seek the presence of conspecifics*, *while unsociable individuals avoid conspecifics*” [13]. Recent studies found that more sociable (assessed through social motivation tests) adult dairy cows [14] and calves [15] spent more time with conspecifics in their home-pen. Here, we explored whether personality traits, such as Fearfulness, Sociability and Pessimism were associated with individual differences in social selectivity and predicted that highly sociable calves would be less selective.

## 2. Material and methods

This study was carried out at the University of British Columbia’s (UBC) Dairy Education and Research Centre, in Agassiz, British Colombia, Canada (49°N, 121°W). The animals were cared for according to the guidelines outlined by the Canadian Council of Animal Care (2009) and all procedures used in this study were approved by the UBC Animal Ethics Committee (#A15-0117).

### 2.1 Animals and housing

This study was conducted using 19 female dairy calves divided in two groups (Group 1: n = 10; Group 2: n = 9) with an average BW at birth of 37.7 ± 4.6 kg raised for the purpose of the UBC dairy education and research center. Within the first 6 hours of life calves received at least 4 L of >50 g/L of IgG colostrum. The umbilical cord was sterilized with a 7% iodine solution immediately following the first colostrum feeding. The calves were housed individually until they were disbudded at 5 d of age using caustic paste and sedation [16]. On the following day, the calves were moved to an open pack pen. The open pack pen measured 35 m^2^ and was equipped with two feeders providing free access to water, hay and calf starter (Hi-Pro Medicated Calf Starter, Chilliwack, BC). The lying area in the pen consisted of an open bedded surface covered with at least 10 cm of sawdust and provided on average 2.6 m² lying space per calf. Calves were weaned at 91 d of age and data collection in the open pack started when the calves were 120 ± 8.3 d of age. Then, the calves were moved to a free stall pen that measured 65 m² (i.e. providing 6.8 m² per calf). The new pen consisted of 13 individual free stall lying spaces (1.44 m² per stall) bedded with washed river sand and a feed bunk with 16 headlocks. Calves were provided hay and total mixed ration (TMR) *ad libitum* and had free access to water. At the end of the experiment, calves were kept for the purpose of the UBC dairy education and research center.

### 2.2. Behavior recording

**Fig 1:**
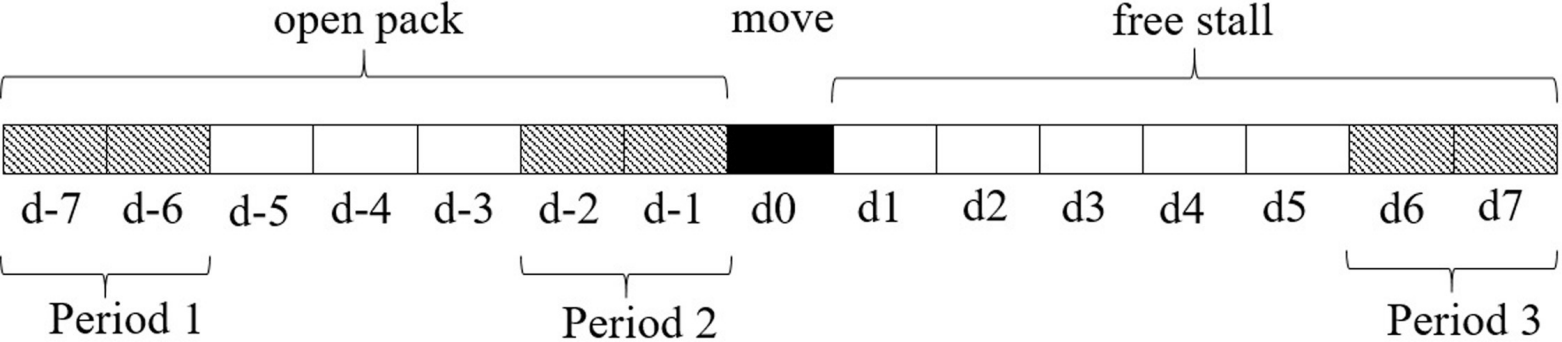
Summary of data collection days in the open pack and free stall pen. Shaded squares indicate days of data collection (5-minute instantaneous scan sampling for 24-h duration). The black square marks the day when dairy calves were moved to the new housing system.

Two cameras (Panasonic, WV-CP310) were placed 6 m above each pen to enable undisturbed behavior observation. As shown in Fig 1, data were collected when animals were in the open pack for 7 d (d -7 to d -1) and in the free stall for 7 d (d 1 to d 7). Calves were video recorded in the open pack for 48 h in two different period (Period 1: day -7 and day -6; Period 2: day -2 and day -1) before moving to the free stall pen (day 0). Calves were again recorded for two consecutive days one week after the move (Period 3: day 6 and day 7). Red lighting above the pens facilitated viewing of calf behaviors during the night.

### 2.3. Behavior assessment

All calves were recognized using their unique fur pattern. Neighbor identity and number of neighbors was assessed using instantaneous scan sampling every 5min (n = 288 scans/calf/day). Most previous studies in cattle assessed the time animals spent in proximity regardless of what they were doing [5,17]. To better understand the situation in which animals are found in close proximity, only occurrences where both animals were lying (i.e. both animals were positioned on the ground with either belly or side of body touching the floor) or standing (i.e. both animals were standing with all four feet on the ground) as neighbors were used to assess social proximity. Animals in physical contact or less than one head length apart were recorded as ‘neighbors’ regardless of orientation [7]. Observations at the feeder were not included as these may also have been associated with competitive interactions over food. In the absence of position recording transponders or spatial proximity loggers, instantaneous scan samplings seemed to confer the best compromise between accuracy of the data collected and time needed to collect the data.

### 2.4. Personality traits

The methodology used to assess Pessimism, Fearfulness and Sociability can be found in Lecorps et al. [18]. A judgment bias test using a spatial learning task similar to that used on sheep [19] was conducted at 25 and 50 d of age. In both sessions, an empty bottle was placed at three ambiguous locations located between the previously rewarded and punished training locations. Given that calves were found consistent over time in their responses [18], Pessimism was calculated using the average latency to reach ambiguous locations on both sessions. In addition, four standardized tests (Open field, Novel object, Human reactivity and Social motivation test) were used to assess Fearfulness and Sociability. These tests are commonly performed in farm animals to describe personality traits [20]. One test was performed per day and were repeated in two sessions of testing (Session 1 started on day 30 and Session 2 on day 53). Personality data used in this study come from data collected for the purpose of a previous study [18]. Calves were found highly consistent in their response to the different personality tests and individual differences could be described as two main dimensions: Fearfulness and Sociability.

### 2.5. Statistical analysis

All analyses were conducted with R (version 3.2.2; R core team, 2017). Pairs frequency matrices were built separately for lying and standing, and the number of times a pair was observed as neighbors was averaged across d-7 and d-6 (Period 1) as well as d-2 and d-1 (Period 2) in the open pack and across d-6 and d-7 (Period 3) in the free stall design. We calculated Pair selectivity scores using Eq 1.

$$\text{Pair}\;\left(\text{A},\text{B}\right)\;\text{Selectivity}\;\text{score}\left(\text{\%}\right)=\frac{Interactions\left(A,B\right)}{\left(All\;Interactions\;\left(A\right)+All\;Interactions\;\left(B\right)\right)/2}\;*100$$ Eq 1

Pair selectivity scores were calculated for each pair and for each period. Linear regression was used to assess consistency over time (i.e. Period 1 and 2), across contexts (i.e. between the open pack and the free stall design), and among behaviors (lying and standing). For consistency across contexts we averaged pairs proximity scores during Periods 1 and 2 in the open pack (separately for standing and lying) and compared these with Period 3 in the free stall pen.

At the individual level, for each focal calf, we calculated individual selectivity scores for each pair a calf was involved in using Eq 2.

$$\text{Individual}\;\text{Selectivity}\;\text{score}\;\text{for}\;\text{Calf}\;\text{A}\;\text{in}\;\text{Pair}\;\left(\text{A},\text{B}\right)=\frac{Interactions\;\left(A,B\right)}{All\;Interactions\;\left(A\right)}\;*100$$ Eq 2

We then calculated the standard deviation of all individual selectivity scores for each pair a calf was involved in. This measure allowed us to discriminate animals according to how variable (i.e. selective) they were. Low variability meant calves tended to equally interact with all the other calves present in the group; whereas, high variability meant calves had unequal relationships with the other calves (i.e. formed some highly selective relationships). We used linear regressions (with calves as the statistical units) to assess whether scores on different personality traits (Sociable, Fearful or Pessimistic) affected how selective a calf was in its relationships. Normality of the residuals and outliers were graphically checked for all models. When normal distribution was not achieved, p-values were calculated via permutation (Package pgirmess).

## 3. Results

**Fig 2:**
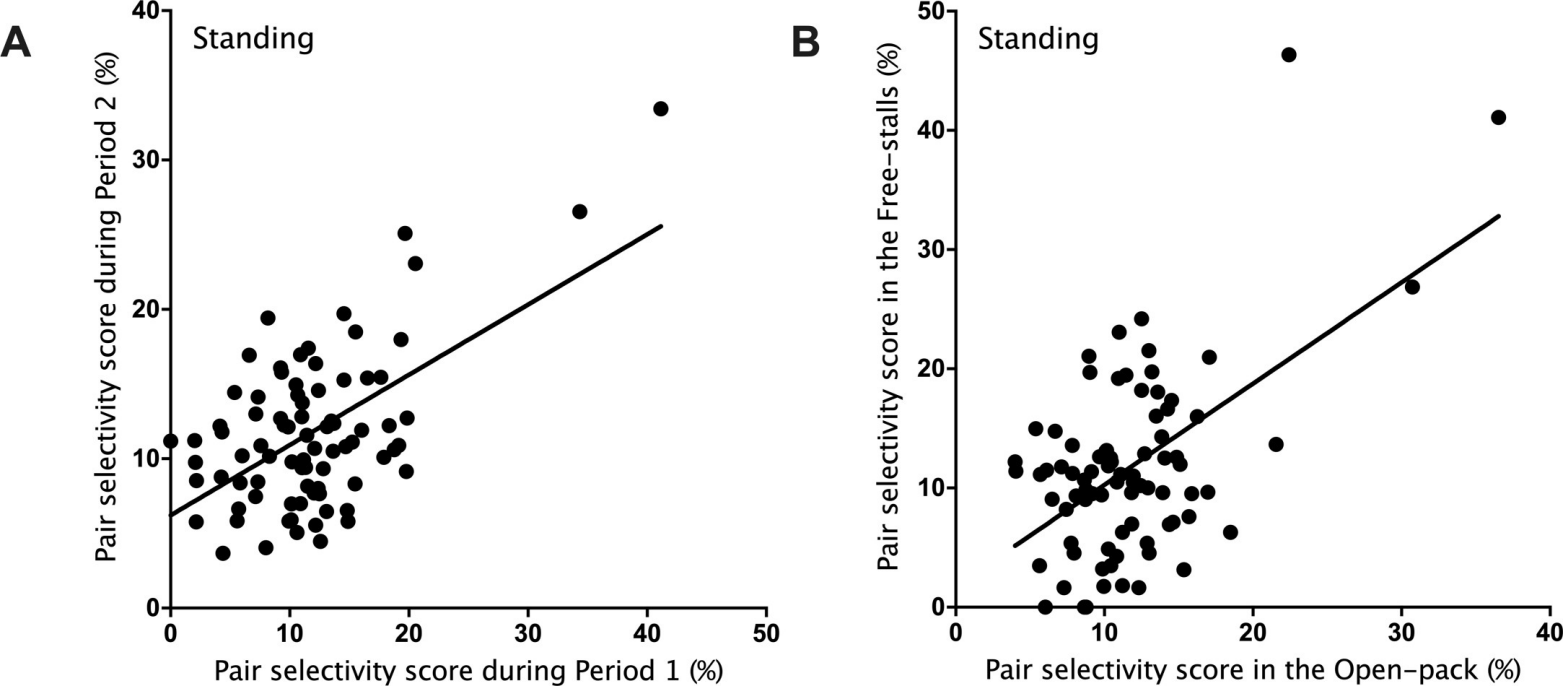
Consistency over time (A) and across contexts (B) for pair selectivity when standing. For each pair, selectivity scores were calculated as the percentage of interactions within the pair relative to the total number of interactions each calf had with all other calves (Eq 1).

Pairs showed little consistency in proximity when lying (*R*^2^ = 0.044, *P* = 0.06; see S1 File) but were more consistent when standing (*R*^2^ = 0.32, *P* < 0.001; Fig 2A), although it is clear that only a few pairs were consistently interacting at high frequencies. There was also little evidence of consistency across behaviors (*R*^2^ = 0.082, *P* = 0.010; see S2 File). Pairs showed little evidence of consistency in lying together before and after the change in housing (*R*^2^ = 0.008, *P* > 0.05; see S3 File), but showed clear evidence of consistency when standing (*R*^2^ = 0.30, *P* < 0.001; Fig 2B). Once again, this result was mostly driven by a few pairs that consistently interacted at higher frequencies.

**Fig 3:**
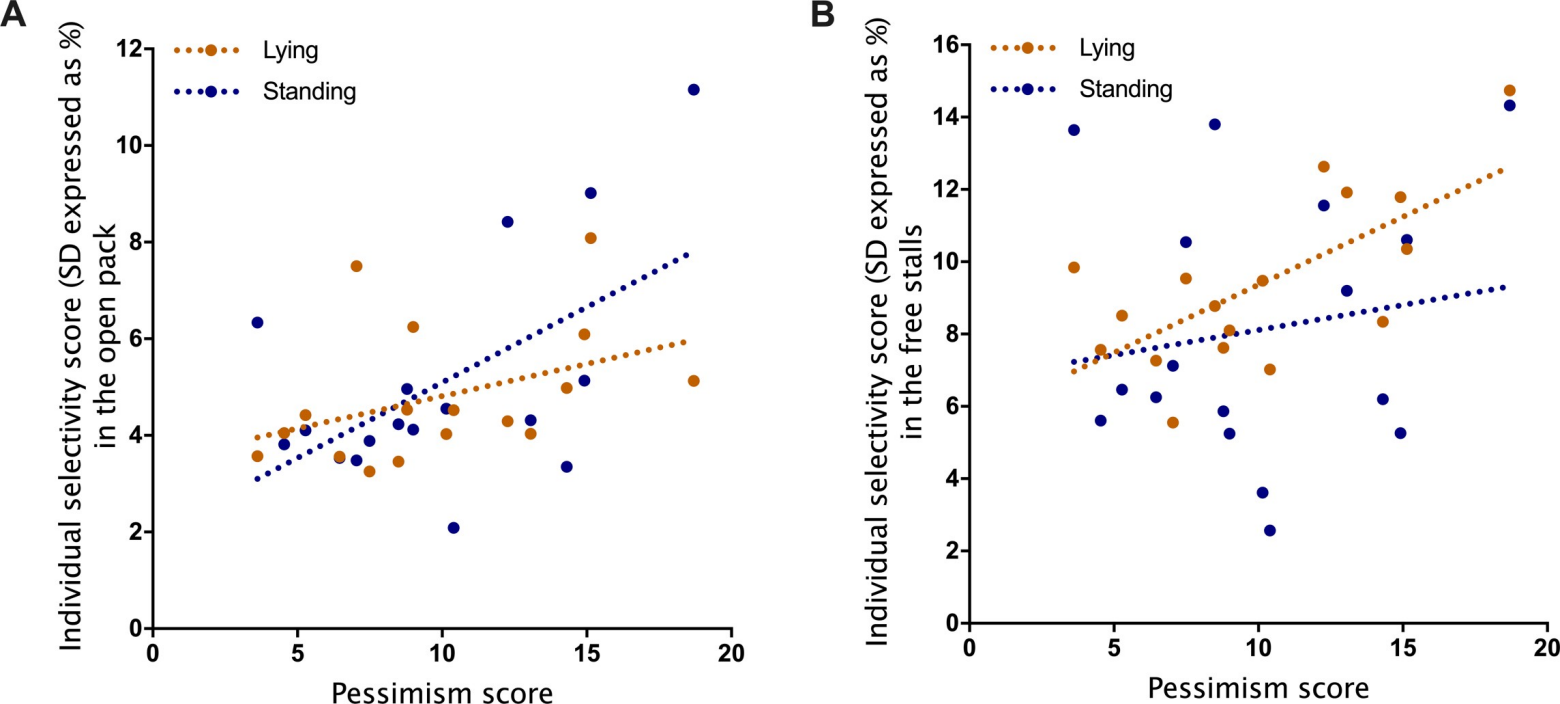
Relationship between pessimism and individual selectivity scores calculated when animals were in the open pack (A) and in the free stalls (B). Selectivity scores and linear regressions are reported for when animals were either lying (orange dots) or standing (blue dots) (n = 18). Selectivity scores were calculated by reporting the number of interactions the individual had with each pair to its total number of interactions. We then calculated the standard deviation of these selectivity scores to get a single selectivity score per individual per observation area (open pack or free stalls) and per behavior (i.e. lying or standing).

Sociability was not related to calves’ selectivity for social partners while lying or standing either in the open pack or the free stalls (all *R*^2^ < 0.05, *P* > 0.05). Similarly, fearfulness was not explaining calves’ selectivity (all *R*^2^ < 0.1, *P* > 0.05) except when calves were lying in the open pack, where a tendency was found (*R*^2^ = 0.18, *P* = 0.09). In contrast, pessimism was positively related to selectivity for social partners when calves were standing in the open pack (*R*^2^ = 0.31, *P* = 0.02; Fig 3A) and tended to be related when calves were lying in the open pack (*R*^2^ = 0.16, *P* = 0.11; Fig 3A). In the free stalls, pessimism explained individual differences in selectivity when calves were lying (*R*^2^ = 0.46, *P* = 0.003; Fig 3B) but not when they were standing (*R*^2^ < 0.05, *P* > 0.05; Fig 3B).

## 4. Discussion

Our results indicate that some pairs of calves interacted more frequently and that this finding was stable over multiple observational periods and across different housing systems. These preferential relationships were only present when the animals were standing. Some calves were more selective than others in their social relationships and these inter-individual differences in social selectivity were partly explained by differences in Pessimism and Fearfulness but not Sociability. More pessimistic calves were more selective in their social relationships.

Overall, consistency of pair formation was greater when calves were standing versus lying. The greater stability of preferential partners when animals were standing is likely associated with the fact that calves could move around, allowing them to easily approach a preferred calf or avoid a less preferred one. Moreover, given calves usually spend more than 14 h/d lying [21], social preference during resting may be highly influenced by a few long-lasting lying bouts. Proximity while lying may have been limited by the design of the free stall only allowing a maximum of two possible neighbors. It is possible that the lying spaces adjacent to the preferred partner may have been already occupied leaving the focal calf no choice but to lie next to a less preferred partner.

Our results are in accordance with previous studies [8,17,22,23], and support the idea that some dairy calves develop preferential bonds with peers at an early age. However, as only a few pairs established highly preferential relationships, our results highlight that calves differed in how they distributed their interactions with the other members of the herd and that not all calves engage in preferential relationships. Although maintenance of social proximity seems a reliable indicator of social bonding [4,5,11,12], future studies should consider the use of more detailed analyses including affiliative behaviors such as grooming events [24]; grooming being a key marker of social bonding in other species [25].

Our results show that only some individuals exhibited highly selective relationships. Why some individuals form more selective relationships is unknown. Previous work showed that the time spent in isolation before being grouped (it is a common practice in the dairy industry to house calves individually during the milk feeding period), can affect the ability to form selective relationships [17]. However, the calves in this study had lived in the same social group since they were 6 days old. Previous research reported that highly sociable dairy calves [15] and dairy cows [14] have greater social proximity scores, indicating that they interact with more animals. Consequently, we expected that highly sociable animals would be less selective in their social relationships explaining why they have more global interactions, but we failed to show any relationships. Our results suggest that social bonding does not depend upon Sociability that probably affects the quantity [15] rather than the specificity of the social relationships.

Although Sociability was not related to individual selectivity, we found more pessimistic, and to lesser extent Fearful animals, displayed higher selectivity in their social relationships. Only a few studies have reported the effects of inter-individual differences in Optimism/Pessimism or Fearfulness in social relationships in animals. For instance, Clegg et al. [26] found more socially synchronized dolphins to make more optimistic choices; whereas, more submissive common marmosets were found more pessimistic [27]. However, in most studies it is hard to know whether Optimism affected the social relationship or vice-versa. It is possible that being submissive leads to negative affective states and induces negative cognitive bias [28]. In humans, Optimism is associated with better social support and a greater social network [29], but it has been argued that having a higher perception of social support could also increase Optimism. In our study, calves were described for Optimism/Pessimism long before their social relationships were observed, making it unlikely that social support affected their response to the judgment bias tests. Although high levels of pessimism did not affect calves’ overall number of social interactions [15], it led to more selective relationships with few members of the social group. These results suggest that pessimistic calves had a smaller social network maybe because they avoided animals that could represent a potential threat. In contrast, higher levels of optimism might be linked to social dominance [27,30] as establishing social dominance requires, at least in part, frequent social contacts with other members of the herd. This might explain why optimistic calves had lower selectivity (better distribution of their social interactions). It remains to be shown that pessimistic dairy calves have lower social ranks, but if true, this lack of social support would add to the growing body of literature suggesting that pessimistic animals might be overly more vulnerable [15,31].

The relationships between pessimism and selectivity scores depended on the environment. Pessimism seemed to explain calves’ selectivity when standing in the open pack but not in the free stalls. In contrast, pessimism was only marginally explaining calves’ selectivity when lying in the open pack but strongly affected it in the free stalls. It seems that the environment had a strong effect on calves’ social behaviors. The open pack offered little possibilities to avoid specific animals when lying because of the restricted space given but easily allowed avoidance behaviors when standing (food being distributed *ad libitum* along the day, calves could feed when they wanted). In the free stall design, the situation was different as feed was only provided twice per day and free stalls offered very selective and protective lying spaces. It was then probably easier for pessimistic calves to be selective in their social relationship when lying. How personality traits interact with the environment and drive social behaviors needs to be further explored as this could explain animals’ preference for specific features of their environment.

The current study was constrained by the limited information obtained using spatial proximity data. Future studies should investigate the relationship between social behaviors and personality traits using more in-depth behavioral analyses and/or different experimentally induced social situations such as dyadic encounters, social isolation and social mixing.

## 5. Conclusion

Spatial proximity in dairy calves illustrates consistent social preferences over time and across contexts for observations when calves were standing, but not for observations based on when calves were lying down. Sociability did not affect individual levels of selectivity but Pessimism and Fearfulness did. More pessimistic and fearful calves seemed to form more selective relationships.

## Supporting information

S1 File Consistency over time between period 1 and 2 for pair selectivity scores while lying. (TIFF)

S2 File Consistency across behaviors (between lying and standing) for pair selectivity scores averaged over period 1 and 2. (TIFF)

S3 File Consistency across contexts (between open-pack and free stalls) for pair selectivity scores while lying. (TIFF)

S4 File Data collected for the experiment. (XLSX)
