# Supplementary figures and images for: Social proximity in dairy calves is affected by differences in pessimism
Source: PLoS One. 2019 Oct 30;14(10):e0223746. doi: 10.1371/journal.pone.0223746 (PMC6821061; doi:10.1371/journal.pone.0223746)

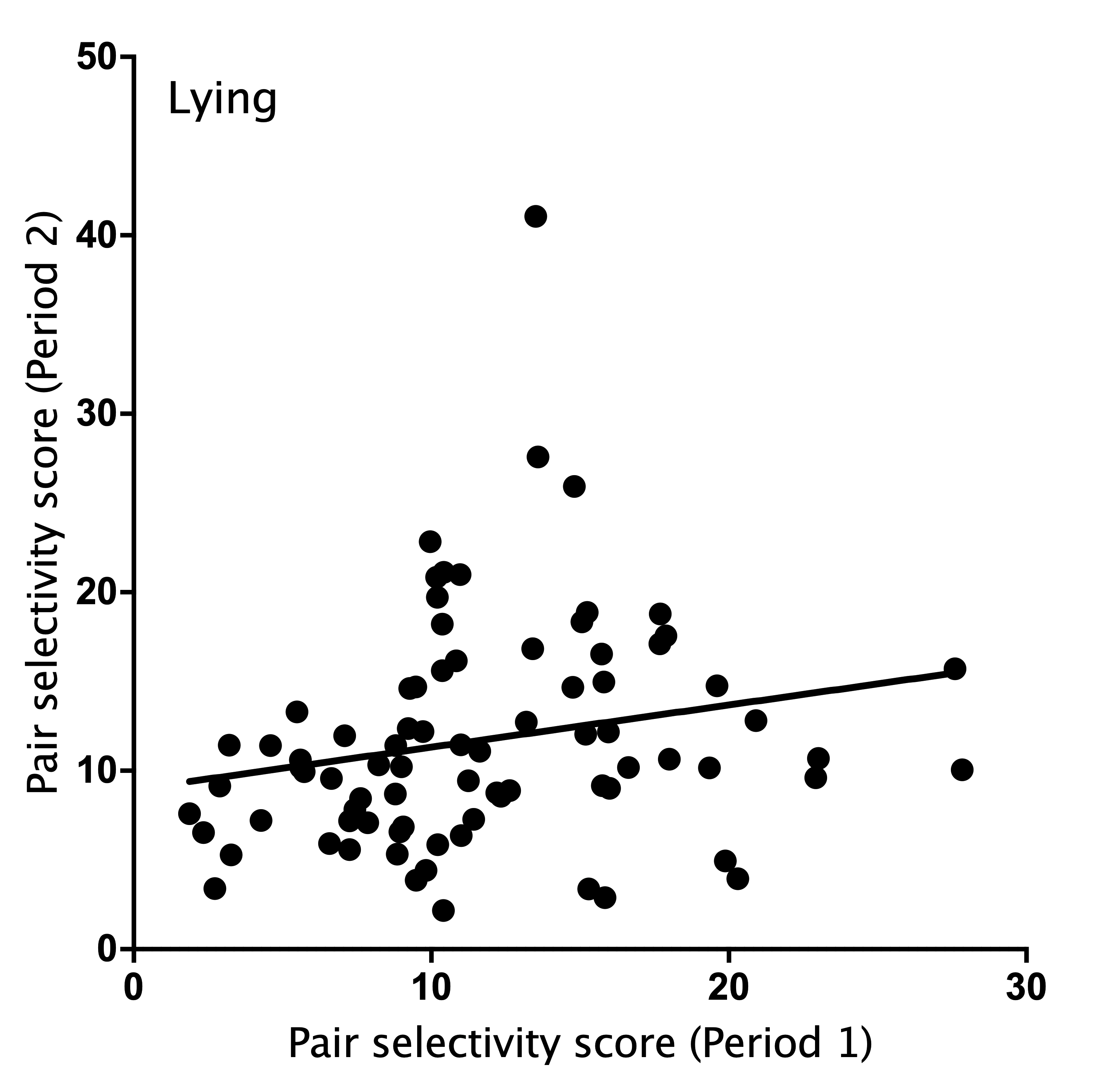

Supplement: S1 File — (TIFF) [file pone.0223746.s001.tiff]

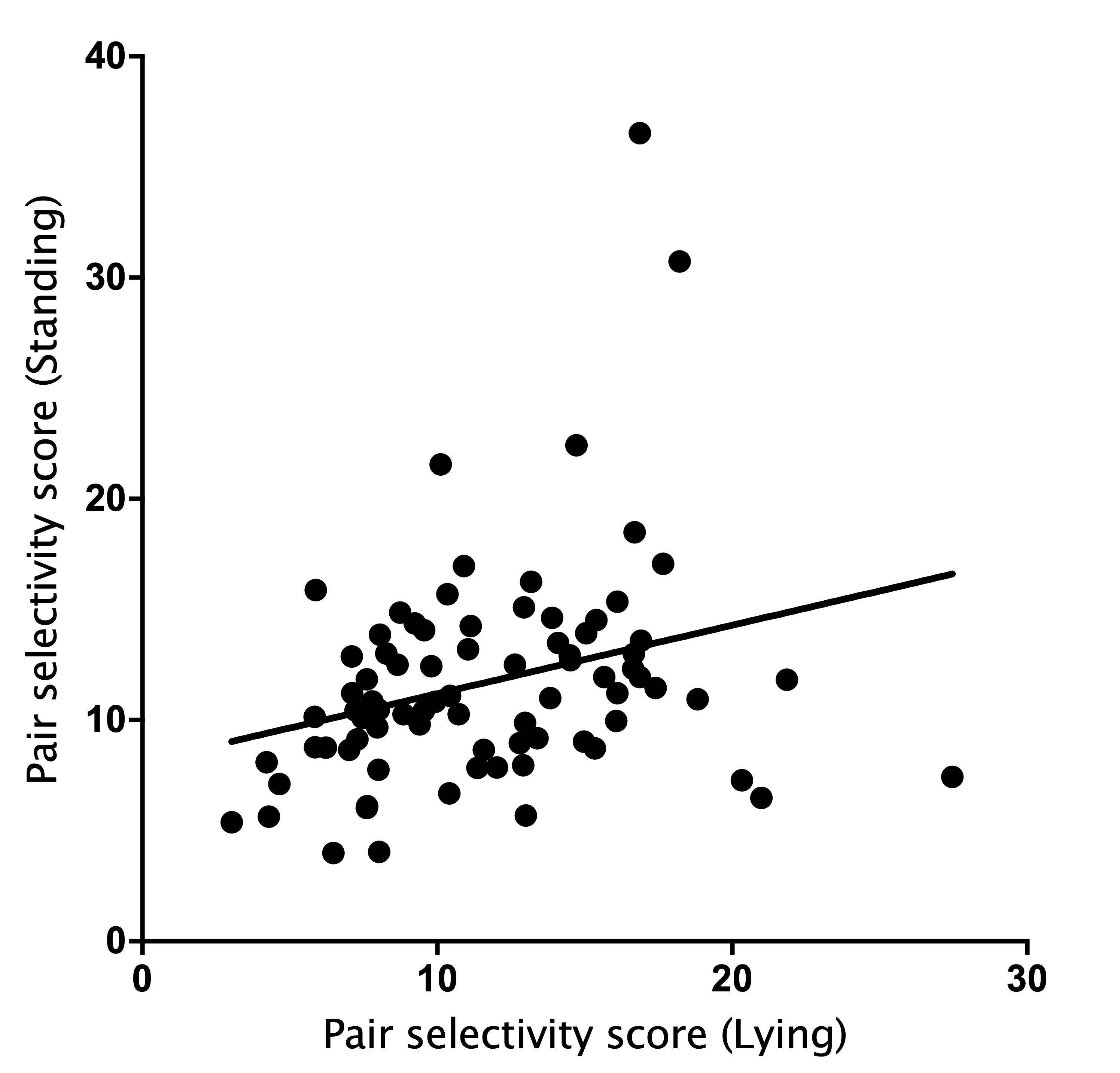

Supplement: S2 File — (TIFF) [file pone.0223746.s002.tiff]

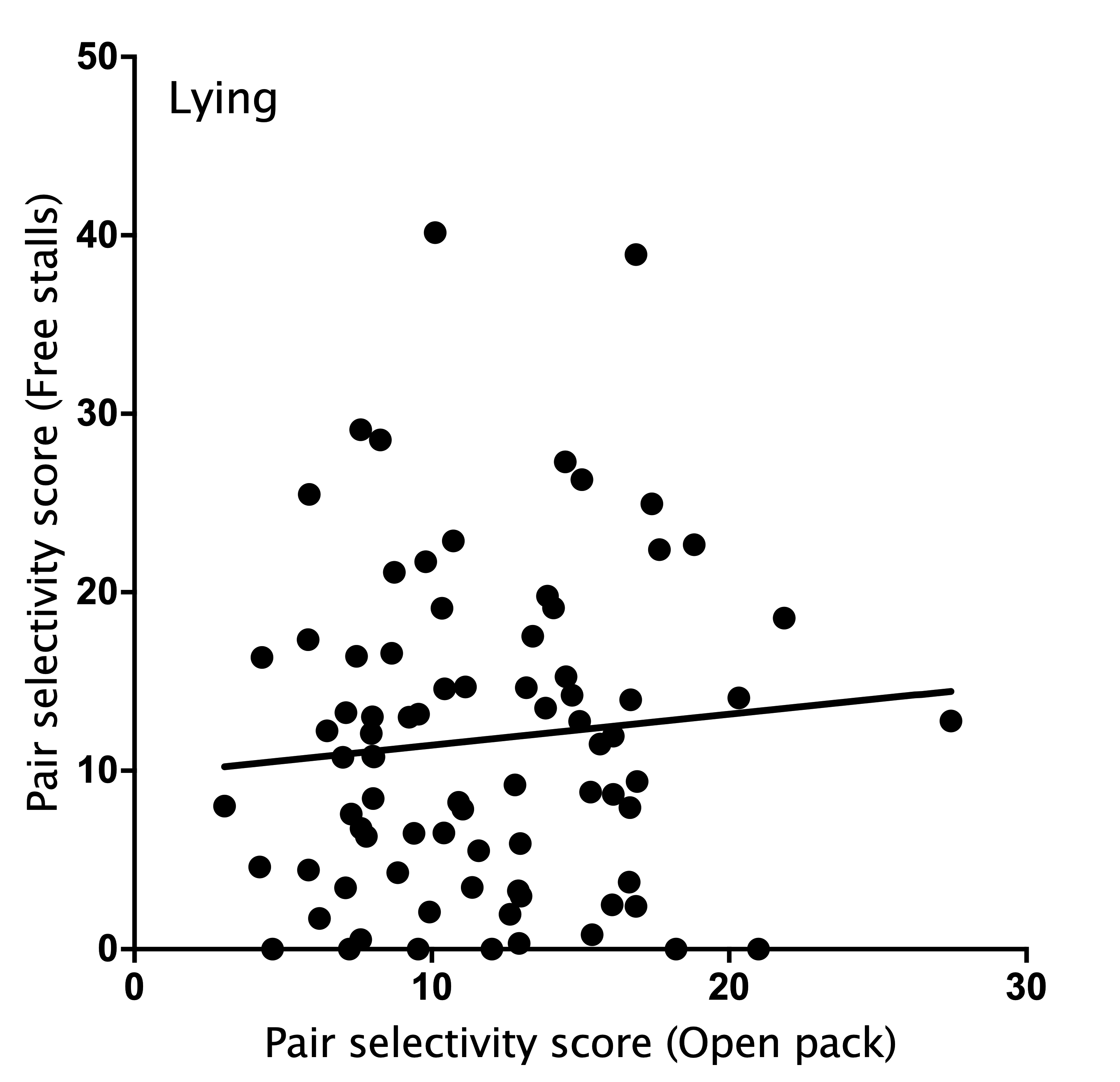

Supplement: S3 File — (TIFF) [file pone.0223746.s003.tiff]
